# Safety, efficacy, and angiographic outcomes of endovascular coiling for very small unruptured intracranial aneurysms (≤ 3 mm): subgroup analysis of the ECOSA registry

**DOI:** 10.3389/fneur.2026.1916793

**Published:** 2026-09-04

**Authors:** Yazan Ashouri, Zain Tariq, Mohammad AlMajali, Mustafa Abdul Kareem, Thanh N. Nguyen, Mo'ath Alsarhan, Amit Chaudhari, Darwin Ramirez-Abreu, Anjan Bhattarai, Raul G. Nogueira, Elad I. Levy, Andrew Xavier, Italo Linfante, Rishi Gupta, Daniel P. Hsu, Jacques E. Dion, C. Michael Cawley, Frank C. Tong, Nils Mueller, Randall C. Edgell, Alexander Norbash, Osama O. Zaidat

**Affiliations:** 1 Endovascular Neurology and Neuroscience, St. Vincent Mercy Medical Center, Toledo, OH, United States; 2 Department of Neuroendovascular Surgery, Ochsner Lafayette General Medical Center, Lafayette, LA, United States; 3 Department of Radiology, Boston Medical Center, Boston, MA, United States; 4 Department of Neurology and Neurosurgery University of Pittsburg Medical Center, UPMC Stroke Institute, Pittsburgh, PA, United States; 5 Department of Neurosurgery, University of Buffalo, Buffalo, NY, United States; 6 Department of Neurology, Wayne State University, Detroit, MI, United States; 7 Department of Interventional Neuroradiology, Baptist Health Neuroscience Institute, Miami, FL, United States; 8 Wellstar Medical Group Neurosurgery, WellStar Medical Group, Marietta, GA, United States; 9 Kaiser Permanente Redwood City Medical Center, Redwood City, CA, United States; 10 Department of Radiology and Neurosurgery, Emory University, Atlanta, GA, United States; 11 Advanced Neuroscience Network, Tenet South Florida, Boyton Beach, FL, United States; 12 Department of Neurology, Saint Louis University, St. Louis, MO, United States

**Keywords:** balloon-assisted coiling, endovascular coiling, ISUIA, natural history, PHASES score, Stent-assisted coiling, unruptured aneurysm, very small intracranial aneurysms

## Abstract

**Introduction:**

Very small unruptured intracranial aneurysms (VSUIAs) measuring ≤ 3 mm represent a unique clinical challenge, balancing the low but non-negligible lifetime risk of rupture against the procedural risks of endovascular intervention. This study reports the safety, efficacy, and angiographic outcomes of endovascular coiling for VSUIAs from the ECOSA (Endovascular Coiling of Small Aneurysms) multicenter registry.

**Methods:**

We performed a subgroup analysis of the ECOSA multicenter registry of patients with VSUIAs (maximum diameter ≤ 3 mm) who underwent endovascular coiling. Patient demographics, aneurysm characteristics, procedural details, periprocedural complications, clinical outcomes (modified Rankin Scale; mRS), and angiographic follow-up data were analyzed.

**Results:**

A total of 116 patients with 116 VSUIAs underwent endovascular coiling. The mean age was 52.3 years (SD 12.1), and the majority were female (75.0%). The mean aneurysm diameter was 2.66 mm (SD 0.44) and mean neck diameter was 2.1 mm (SD 0.87). Most aneurysms were discovered incidentally (78.4%). Balloon-assisted coiling was used in 14.7% and stent-assisted coiling in 23.3% of cases. The thromboembolic event (TEE) rate was 1.7%, with no symptomatic events. Intraprocedural rupture occurred in 0.9% of cases with no intraprocedural mortality. Among 98 patients with clinical follow-up (median 7.75 months), 93.9% of patients achieved good functional outcomes (mRS 0–2), and all-cause mortality rate was 1.0%. Among 80 patients with angiographic follow-up (median 6 months), adequate occlusion (RROC I–II) was achieved in 86.3%.

**Discussion:**

Endovascular coiling of VSUIAs ≤ 3 mm is feasible and associated with a low complication rate, high rates of good functional outcomes, and durable angiographic occlusion. These findings support the safety of elective intervention in carefully selected patients with high-risk morphological or clinical features. Larger prospective studies are needed to define optimal patient selection criteria for VSUIAs.

## Introduction

Intracranial aneurysms are identified in approximately 2–5% of the general population, with the widespread adoption of cross-sectional neuroimaging leading to increasing detection of incidentally discovered lesions, particularly those of very small size (1, 2). Very small unruptured intracranial aneurysms (VSUIAs), commonly defined as those measuring ≤ 3 mm in maximum diameter, represent a distinct subgroup that can be a management dilemma. On the one hand, aneurysmal subarachnoid hemorrhage (SAH) carries a high morbidity and mortality rate; on the other hand, the annual rupture risk is generally reported to be low, and the technical challenges of endovascular therapy can be noted due to the thin fragile wall of those aneurysms and limited space for microcatheter or coil manipulation (3–5).

The natural history of unruptured intracranial aneurysms has been characterized by landmark studies including the International Study of Unruptured Intracranial Aneurysms (ISUIA), and the Unruptured Cerebral Aneurysm Study (UCAS) (6, 7). The ISUIA reported an annual rupture risk of 0.1% for anterior circulation aneurysms smaller than 7 mm in patients without a prior history of SAH, and 0.5% in those with a history of prior SAH (6). The PHASES score, which incorporates population, hypertension, age, aneurysm size, earlier SAH, and aneurysm site, provides a widely used multivariable framework for rupture risk estimation, though size remains the dominant predictor (8). However, emerging evidence suggests that aneurysm size alone is an insufficient basis for management decisions, and that aneurysm morphology significantly modifies individual rupture risk and may shift the risk-benefit ratio toward intervention even for small aneurysms (9, 10).

The small aneurysm sac limits the number and size of coils that can be safely deployed, relatively increasing the risk of intraprocedural rupture and thromboembolic events (TEE). Earlier meta-analyses of coiling for aneurysms ≤ 3 mm reported procedural rupture rates as high as 8.3% and combined morbidity/mortality rates of approximately 4% (11). However, advances in coil technology, microcatheter design, and operator experience, as well as balloon- and stent-assisted techniques, may have improved outcomes in practice (12).

Real-world data on the safety and efficacy of endovascular coiling specifically in this size remain limited, with most published series dominated by ruptured cases. This study reports the periprocedural safety, functional outcomes, and angiographic results of endovascular coiling for VSUIAs ≤ 3 mm from a retrospective multicenter registry.

## Materials and methods

### Study design and patient population

The Endovascular Coiling of Unruptured Small Aneurysm (ECOSA) study group is a retrospective analysis of clinical outcomes for patients undergoing endovascular coiling aneurysm in 13 centers in the United States. Patients were included in the ECOSA registry if they had an unruptured aneurysm smaller than 7 mm, whereas the present subgroup analysis only included aneurysms ≤ 3 mm in the maximum diameter. Patients with previous (but not acute) subarachnoid hemorrhage presenting for elective coiling were included. We collected demographics, location, size of the aneurysm, and peri-procedural complications. The study was determined to be exempt from Institutional Review Board (IRB) approval due to retrospective design and presentation of deidentified aggregate data.

### Endovascular procedure

All procedures were performed by experienced neurointerventionalists under general anesthesia using standard endovascular techniques. Transfemoral arterial access was used in all cases. Simple coiling was performed when the aneurysm anatomy was favorable; balloon-assisted coiling (BAC) was employed in cases where the neck-to-dome ratio necessitated remodeling to prevent coil herniation, and stent-assisted coiling (SAC) was used for wide-necked aneurysms where balloon remodeling was deemed insufficient. Antiplatelet premedication was administered at operator discretion and per institutional protocol, typically consisting of dual antiplatelet therapy (aspirin and clopidogrel) for patients undergoing SAC. Intravenous heparin was administered in all cases, but the timing (immediately before, during, or after the procedure) varied according to operator preferences or institutional protocols.

### Outcome measures

The primary safety outcomes were periprocedural thromboembolic events (TEE), intraprocedural rupture and intraprocedural mortality. Clinical outcomes were assessed using the modified Rankin Scale (mRS) at last follow-up, with good functional outcome defined as mRS 0–2. Angiographic outcomes were assessed using the Raymond-Roy Occlusion Classification (RROC): RROC I (complete occlusion), RROC II (neck remnant), and RROC III (residual aneurysm). Adequate occlusion was defined as RROC I or II. Follow-up imaging was performed by digital subtraction angiography (DSA), magnetic resonance angiography (MRA), or computed tomography angiography (CTA) at operator and institutional discretion.

### Statistical analysis

Continuous variables were summarized as mean with standard deviation (SD), and categorical variables as frequencies and percentages. Percentages were based on the number of patients with available data for each variable, and clinical and angiographic outcomes were assessed among patients with available follow-up. Functional outcome was graded using the modified Rankin Scale (mRS), with mRS 0–2 defined as a good outcome, and angiographic occlusion using the Raymond–Roy Occlusion Classification (RROC). All analyses were performed using IBM SPSS Statistics for Windows, version 28.0 (IBM Corp, Armonk, NY).

## Results

## Patient and aneurysm characteristics

A total of 903 subjects with small ( ≤ 7 mm) unruptured intracranial aneurysms were consecutively enrolled in the ECOSA registry. Among these, 116 subjects harbored very small ( ≤ 3 mm) unruptured aneurysms. The patient characteristics and demographics are outlined in Table 1. The mean patient age was 52.3 years (SD 12.1), with a predominance of female patients (87/116, 75.0%). Most patients were white (71/116, 61.7%). Hypertension was prevalent in 61.2%, diabetes mellitus in 6.9%, and coronary artery disease in 10.3%. Almost one third of patients were current smokers (37/116, 31.9%) or former smokers (35/116, 30.2%). A family history of an intracranial aneurysm was identified in 17 patients (14.7%).

**Table 1:** Patient demographics.

Variable, *n* (%)	*n* (%), *N* = 116 subjects
Age, mean (SD), year	52.3 (12.1)
Female	87 (75.0%)
Race	
Caucasian/White	71 (61.7%)
African American/Black	33 (28.4%)
Other	11 (9.5%)
Comorbidities	
Hypertension	71 (61.2%)
Diabetes Mellitus	8 (6.9%)
Previous smoker	35 (30.2%)
Current smoker	37 (31.9%)
Coronary Artery Disease	12 (10.3%)
Polycystic Kidney Disease	3 (2.6%)
Cocaine abuse	6 (5.2%)
Family history of aneurysm	17 (14.7%)
Aneurysm site	
Acomm/ACA	25 (21.7%)
Pcomm	10 (8.7%)
Basilar artery	11 (9.5%)
ICA	42 (36.2%)
MCA	15 (12.9%)
VBA (except basilar tip)	17 (14.7%)
Other	4 (3.4%)
Indication for Coiling	
Incidental	91 (78.4%)
Mass Effect	5 (4.3%)
Old SAH	15 (12.9%)
Distal Embolization	3 (2.6%)
Other	2 (1.7%)
Shape of Aneurysm	
Spherical	2 (2.6%)
Oblong/Ovoid	10 (8.6%)
Irregular with daughter sac	14 (12.1%)
Bilobed	7 (6.0%)
Triangular	1 (0.9%)
Saccular	28 (24.1%)
Other	2 (1.8%)
Aneurysm max diameter, mean (SD)	2.66 (0.44)
Neck diameter, mean (SD)	2.1 (0.87)

Acomm, anterior communicating artery; ACA, anterior cerebral artery; Pcomm, posterior communicating artery; ICA, internal carotid artery; MCA, middle cerebral artery; VBA, vertebrobasilar; SAH, subarachnoid hemorrhage; SD, standard deviation.

**Table 2:** Procedural and clinical outcomes.

Variable, n (%)	n (%), N = 116 subjects
Balloon assisted coiling	17 (14.7%)
Stent assisted coiling	27 (23.3%)
Thromboembolic events	
Total	2 (1.7%)
Symptomatic	2 (1.7%)
Intraprocedural rupture	1 (0.9%)
Intraprocedural mortality	0 (0.0%)
Clinical follow-up (*N* = 98)	
Time to follow-up, median (IQR), months	7.75 (1–8.8)
mRS 0–2	92 (93.9%)
All-cause mortality	1 (1.0%)
Angiographic follow-up (*N* = 80)	
Time to follow-up, median (IQR), months	6 (2.8–10.6)
Imaging technique	
DSA	33 (41.3%)
MRA	31 (38.8%)
CTA	6 (7.5%)
Unspecified	10 (12.5%)
Angiographic occlusion	
RROC I	59 (73.8%)
RROC II	10 (12.5%)
RROC I-II	69 (86.3%)
RROC III	11 (13.8%)

mRS, modified Rankin scale; DSA, diagnostic angiogram; MRA, Magnetic resonance angiography; CTA, computed tomography angiography; RROC, Raymond Roy occlusion.

The most common indication for treatment was an incidentally discovered aneurysm during workup for headaches (78.4%), followed by remote history of SAH from a previously ruptured aneurysm (12.9%). Locations included the internal carotid artery (ICA) in 42 patients (36.2%), the anterior communicating artery/anterior cerebral artery (Acomm/ACA) complex in 25 (21.7%), the vertebrobasilar arteries (excluding the basilar tip) in 17 (14.7%), the middle cerebral artery (MCA) in 15 (12.9%), the basilar artery in 11 (9.5%), and the posterior communicating artery (Pcomm) in 10 (8.7%). Aneurysms were most of saccular morphology (24.1%), or irregular with daughter sac (12.1%). The mean maximum aneurysm diameter was 2.66 mm (SD 0.44) and mean neck diameter was 2.1 mm (SD 0.87).

Primary coiling was performed in the majority of cases. Balloon-assisted coiling (BAC) was utilized in 17 patients (14.7%), and stent-assisted coiling (SAC) in 27 patients (23.3). Technical success was achieved in all cases.

Thromboembolic events (TEE) occurred in two patients (1.7%), neither of which was symptomatic. An intraprocedural rupture occurred in 1 patient (0.9%). There was no intraprocedural mortality.

## Clinical outcomes

Clinical follow-up was available for 98 patients at a median of 7.75 months (IQR 1–8.8 months). Of these, 93.9% achieved a good functional outcome (mRS 0–2). All-cause mortality during follow-up occurred in one (1.0%) patient. No cases of aneurysm rupture or bleeding were documented during the follow-up period.

## Angiographic Outcomes

Angiographic follow-up was available for 80 patients at a median of 6 months (IQR 2.8–10.6 months). Imaging modalities included DSA in 33 patients (41.3%), MRA in 31 (38.8%), CTA in six (7.5%), and unspecified in 10 (12.5%). At follow-up, complete occlusion (RROC I) was achieved in 59 patients (73.8%), neck remnant (RROC II) in 10 (12.5%). Adequate occlusion (RROC I–II) was achieved in 69 patients (86.3%).

## Discussion

This study presents the safety, efficacy, and angiographic outcomes of endovascular coiling for VSUIAs ≤ 3 mm from a retrospectively maintained multicenter registry. The current study findings suggest that endovascular coiling of VSUIAs is feasible with a low periprocedural complication rate. Excellent functional outcomes were achieved in 93.9% of patients with available follow-up and adequate occlusion (RROC I–II) was achieved in 86.3% at midterm follow-up.

The natural history of VSUIAs has been characterized by several landmark epidemiologic studies. The ISUIA reported an annual rupture risk of 0.1% for anterior circulation aneurysms smaller than 7 mm in patients without prior SAH history, rising to 0.5% per year for those with prior SAH (6). The UCAS Japan study reported an annual rupture rate of 0.36% for aneurysms 3–4 mm in maximum diameter (7). The SUAVe study, a prospective Japanese cohort of 374 patients with aneurysms < 5 mm, documented a 0.5% overall annual rupture risk, with size >4 mm conferring additional risk (14). The PHASES score which incorporates six independently validated risk factors, estimated a 5-year cumulative rupture risk of approximately 0.4–1.3% for aneurysms ≤ 3 mm size range depending on individual patient characteristics (8). While these aggregate figures appear low, they do not fully capture the disproportionate burden of morbidity and mortality associated with rupture when it does occur, nor the lifetime accumulated rupture risk in younger patients. Furthermore, emerging data suggest that aneurysm morphology may substantially modify rupture risk independent of size (9).

The current study findings compare favorably with both historical and contemporary literature on coiling of VSUIAs. A study by Nguyen et al. reported an intraprocedural rupture rate of 11.7% for ruptured aneurysms ≤ 3 mm treated with endovascular coiling, predominantly in the pre-modern coil technology era (13). A more recent meta-analysis of 42 studies including 2,166 ruptured tiny saccular aneurysms ≤ 3 mm reported a follow-up complete occlusion rate of 83.9%, recanalization rate of 7.7%, and favorable outcome rate of 85.6% (1, 2). Our series, comprising exclusively unruptured aneurysms, demonstrated a substantially lower intraprocedural rupture rate of 0.9%, a TEE rate of 1.7% (with 0% symptomatic events), and a 93.9% rate of good functional outcome.

It is important to mention that the current study included a percentage in which adjunctive endovascular techniques were used (BAC in 14.7% and SAC in 23.3% of patients) which could represent the anatomical complexity of VSUIAs in the current cohort. The 86.3% rate of adequate occlusion at midterm follow-up is consistent with reported rates for contemporary coiling series of small aneurysms and is comparable with flow diversion which may achieve similar complete occlusion rates but require longer times to occlusion and is associated with TEE risks requiring prolonged antiplatelet therapy (15).

Patient selection is a critical determinant in the risk-benefit balance for VSUIAs. Our cohort was enriched for clinical and morphological risk factors that may justify prophylactic treatment despite a small aneurysm size. Hypertension was present in 61.2% of patients, current smoking in 31.9%, and family history of intracranial aneurysm in 14.7%, which are all established independent risk factors for aneurysm rupture (9, 10). Polycystic kidney disease, a condition associated with elevated aneurysm prevalence and rupture risk, was documented in 2.6% of patients. Also, a significant proportion of aneurysms exhibited morphological risk factors for rupture. Additionally, 12.9% of patients had a history of prior SAH from a separately treated aneurysm, a factor that might multiply the rupture rate up to fivefold (6). Taken together, the risk profile of our cohort likely exceeds that of the general population and may account for the clinical decision to treat in these cases. Moreover, as the mean patient age was 52.3 years, it would be reasonable that younger patients have a longer residual lifetime over which the cumulative rupture risk accumulates, and a favorable long-term outcome from prophylactic treatment is more likely to outweigh the one-time procedural risk when life expectancy is considered. A comparative effectiveness analysis by Malhotra et al. (10) demonstrated that coiling becomes the optimal strategy when the annual rupture risk of non-growing aneurysms exceeds 1.7%, a threshold that selected high-risk patients in our cohort may approach given their risk factor burden.

The current study has multiple limitations. The angiographic follow-up rate was 69% (80/116) in our series and a subset of patients had only clinical follow-up without angiographic reassessment. Among those with imaging follow-up, the median interval of 6 months may be insufficient to capture late recanalization (16). Moreover, the heterogeneity in follow-up imaging modalities further limits direct comparisons, as MRA and CTA are known to have lower sensitivity for detecting small residual filling compared to catheter angiography.

Despite these limitations, our data contributes to the growing body of evidence supporting the safety of endovascular coiling for VSUIAs ≤ 3 mm. The low periprocedural complication rates, high rates of good functional outcomes, and durable angiographic occlusion documented in this series suggest that, in carefully selected patients with compounding risk factors, prophylactic endovascular treatment can be performed with an acceptable risk profile that may justify intervention in preference to indefinite surveillance.

## Conclusion

Endovascular coiling of very small unruptured intracranial aneurysms ≤ 3 mm is feasible and safe, with a thromboembolic event rate of 1.7% (0% symptomatic), intraprocedural rupture rate of 0.9%, and good functional outcomes in 93.9% of patients at follow-up. Adequate angiographic occlusion was achieved in 86.3% of patients at midterm imaging follow-up. Larger prospective multicenter registries with standardized imaging follow-up protocols are needed to further define the optimal management strategy for this challenging aneurysm subgroup.

## Data Availability

The original contributions presented in the study are included in the article/supplementary material, further inquiries can be directed to the corresponding authors.
